# Evaluation of radiograph-based interstitial implant dosimetry on computed tomography images using dose volume indices for head and neck cancer

**DOI:** 10.4103/0971-6203.33242

**Published:** 2007

**Authors:** Ritu Raj Upreti, S. Dayananda, R. L. Bhalawat, Girish N. Bedre, D. D. Deshpande

**Affiliations:** Department of Medical Physics, Tata Memorial Hospital, Mumbai, India; * Department of Radiation Oncology, Tata Memorial Hospital, Mumbai, India

**Keywords:** Dose volume indices, head and neck cancer, interstitial implant dosimetry

## Abstract

Conventional radiograph-based implant dosimetry fails to correlate the spatial dose distribution on patient anatomy with lack in dosimetry quality. Though these limitations are overcome in computed tomography (CT)-based dosimetry, it requires an algorithm which can reconstruct catheters on the multi-planner CT images. In the absence of such algorithm, we proposed a technique in which the implanted geometry and dose distribution generated from orthogonal radiograph were mapped onto the CT data using coordinate transformation method.

Radiograph-based implant dosimetry was generated for five head and neck cancer patients on Plato Sunrise treatment planning system. Dosimetry was geometrically optimized on volume, and dose was prescribed according to the natural prescription dose. The final dose distribution was retrospectively mapped onto the CT data set of the same patients using coordinate transformation method, which was verified in a phantom prior to patient study. Dosimetric outcomes were evaluated qualitatively by visualizing isodose distribution on CT images and quantitatively using the dose volume indices, which includes coverage index (CI), external volume index (EI), relative dose homogeneity index (HI), overdose volume index (OI) and conformal index (COIN).

The accuracy of coordinate transformation was within ±1 mm in phantom and ±2 mm in patients. Qualitative evaluation of dosimetry on the CT images shows reasonably good coverage of target at the expense of excessive normal tissue irradiation. The mean (SD) values of CI, EI and HI were estimated to be 0.81 (0.039), 0.55 (0.174) and 0.65 (0.074) respectively. The maximum OI estimated was 0.06 (mean 0.04, SD = 0.015). Finally, the COIN computed for each patient ranged from 0.4 to 0.61 (mean 0.52, SD = 0.078).

The proposed technique is feasible and accurate to implement even for the most complicated implant geometry. It allows the physicist and physician to evaluate the plan both qualitatively and quantitatively. Dose volume indices derived from CT data set are useful for evaluating the implant and comparing different brachytherapy plans. COIN index is an important tool to assess the target coverage and sparing of normal tissues in brachytherapy.

Interstitial implant dosimetry has been conventionally carried out using a set of radiographs which represent the spatial distribution of the implanted catheters within the patient. The correlation of the dose distribution thus obtained with the patient anatomy is relatively subjective and lacks quantitative measure with respect to the target volume coverage. Limited work on three-dimensional computed tomography (3D-CT) based interstitial implant dosimetry demonstrated its potential in providing spatial dose distribution on patient anatomy and quantitative evaluation based on the dose-volume relationship of different anatomical structures.[1–4] However, this method is not always feasible in the absence of a catheter reconstruction algorithm which supports reconstruction on the multi-planar reconstructed CT images. This algorithm is particularly important for complex implants such as head and neck or extremities where the implanted plane is oblique or parallel to the imaging plane and loops are in use. However, this algorithm may not be available in all the commercial treatment planning systems (TPS's) or it comes with an extra cost. To evaluate implant quality based on the dose-volume relation of patient anatomy in the absence of such reconstruction algorithm, we proposed a technique wherein implant geometry was reconstructed from the orthogonal radiograph and the final dose distribution was mapped onto the CT data of the same patient using the coordinate transformation method.

## Materials and Methods

Five patients of various head and neck cancers (tongue, valleculla and epiglottis) were selected for this study. These cases represent complex geometry wherein implanted catheters were oblique and parallel to the imaging plane. The use of loop catheters further complicated the implant geometry and posed difficulties in reconstruction from CT images. All patients had three-plane implant with number of catheters ranging from 9 to 16 (average 11). Catheters in a plane were arranged more or less parallel and equidistant to each other, and loop technique was employed in the posterior-most catheters.

**Figure 1:**
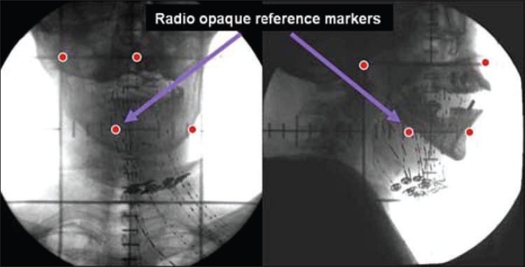
Orthogonal radiographs with X-ray dummy's and radio opaque reference markers

**Figure 2:**
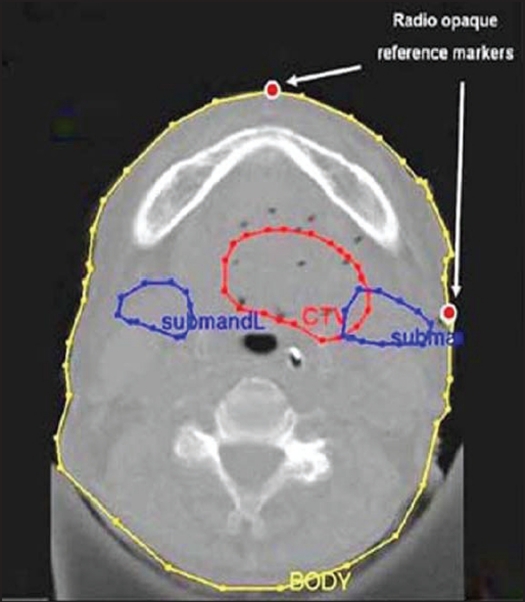
CT image containing the contours and radio-opaque reference markers

Post-implant orthogonal radiographs were taken for each patient on simulator (Ximatron, Varian Medical System, USA) with radio-opaque dummy markers in the implanted catheters [Figure 1]. Reference points were also marked on the patients' skin at different axial levels, and radio-opaque markers were placed prior to acquiring radiographs. Soon after, axial CT images were acquired with 3 mm slice thickness on Somatom Emotion CT scanner (Siemens Medical Systems, Germany) with radio-opaque reference markers in place and dummies removed from the catheters. The target and critical organs were contoured on the axial CT images. Figure 2 represents one of the axial CT images containing the contours and radio-opaque reference markers.

The geometry of the implanted catheters was reconstructed from orthogonal radiographs, and dosimetry was carried out on Plato Sunrise brachytherapy (BT) TPS (Nucletron, Holland) as is usually done for conventional planning. All the reference markers were digitized both in x-ray and CT images. Besides external reference markers, radio-opaque buttons on both ends of the implanted catheters were taken as internal reference markers for the subsequent mapping of x-rays and CT data sets. All catheters were loaded with alternate dwell positions of step size 2.5 mm, leaving 5 to 10 mm from the open end of the tube to minimize skin dose. The treatment plan was geometrically optimized and prescribed on the natural prescription dose (NPD) derived from the Anderson natural dose volume histogram (DVH).[5] The final plan was then superimposed retrospectively onto the CT data set by co-registering the reference radio-opaque markers of the radiograph with the corresponding reference markers on the CT data sets using coordinate transformation method available with Plato Sunrise BT TPS. The accuracy of the coordinate transformation was investigated prior to patient study on a customized phantom. The phantom simulated single-plane implant of predefined geometry consisting of eight flexible catheters and known reference markers.

Qualitative evaluation of the dosimetric outcome was carried out by visualizing the mapped isodose distribution in each slice of CT. Quantitative evaluation of the implant dosimetry was carried out using various indices derived from the dose volume relationship of the patient's CT images. The indices included the coverage index (CI), external volume index (EI), relative dose homogeneity index (HI) and overdose volume index (OI).[6] For quantitative evaluation of conformality, the conformal index (COIN) was used.[7]

CI is the fraction of PTV receiving a dose equal to or greater than the reference dose:

$CI=\frac{PTV_{100}}{V_{PTV}}$

EI is the ratio of the normal tissue volume outside the PTV receiving a dose equal to or greater than the reference dose, to the PTV:

$EI=\frac{\left(V_{100}\text{−}PTV_{100}\right)}{V_{PTV}}$

HI is the fraction of PTV receiving a dose between 100 and 150% of the reference dose:

$EI=\frac{\left(V_{100}\text{−}PTV_{150}\right)}{PTV_{100}}$

OI is the fraction of PTV receiving a dose equal to or greater than two times the reference dose:

$OI=\frac{PTV_{200}}{PTV_{100}}$

The COIN takes into consideration the coverage of PTV by the reference dose and also the unwanted irradiation of normal tissue outside the PTV:

$COIN=\frac{PTV_{200}}{V_{PTV}}\times \frac{PTV_{100}}{V_{100}}$

In the above equations, PTV_100_ is the volume of target receiving a dose equal to or greater than the reference dose, PTV_150_ is the volume of PTV receiving 1.5 times of the reference dose, PTV_200_ is the volume of PTV receiving equal to or greater than two times the reference dose, V_100_ is the volume of tissue that received reference dose and V_PTV_ is the total volume of PTV.

## Results

**Figure 3:**
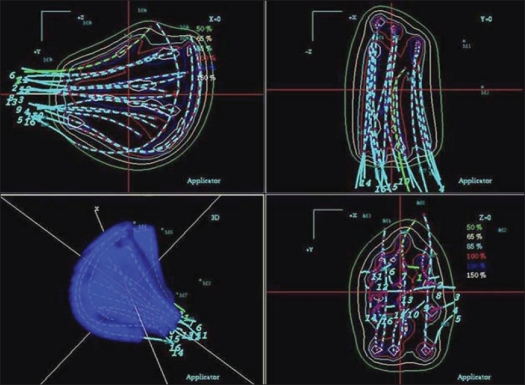
2D and 3D dose distribution resulted from radiograph generated plan

**Figure 4:**
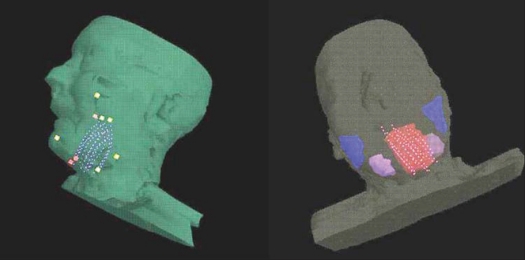
The 3D distribution of reconstructed implant geometry transformed on to the CT dataset

The accuracy of coordinate transformation found in the phantom study was within ± 1 mm. Table 1 shows the variation in the coordinate transformation between orthogonal radiograph and corresponding CT data of the five patients. The goodness of the fit was estimated from chi-square test of transformation matrix. The maximum of the mean variation observed in all patients was 0.97 mm (range 0-0.97 mm). Figure 3 represents the two-dimensional (2D) and three-dimensional (3D) dose distribution resulted from radiograph-based plan. The 3D distribution of reconstructed implant geometry transformed onto the CT data sets is shown in Figure 4, whereas Figures 5A, 5B and 5C represent the mapped dose distribution on the axial, sagittal and coronal planes respectively. The radiograph-based dosimetry was evaluated qualitatively by observing mapped isodoses on each and every CT slice to ensure adequate coverage of the target volume with the reference isodose value. The quantitative evaluation of the dosimetric outcome was derived from the cumulative DVH of the defined structures on the CT data set and is shown in Figure 6. Median volume of CTV measured from CT data set was 42.9 cc (range 38.6-58.3 cc). Table 2 represents the different indices derived from the DVH of each patient. The mean CI was found to be 0.81 (range 0.77-0.87, SD = 0.039). Only in one patient, the estimated EI was less (0.3); while in the remaining patients, EI was more than 0.5. The mean EI for all patients was estimated to be 0.55 (range 0.3-0.76, SD = 0.174). The HI was found to be in range of 0.55-0.74 (mean 0.65, SD = 0.074). The maximum OI estimated was 0.06 (mean 0.04, SD = 0.015). Finally the COIN computed for each patient was in the range of 0.4-0.61 (mean 0.52, SD = 0.078).

**Table 1:** Variation in the coordinate transformation between orthogonal radiograph and corresponding CT data of different patients

*Reference markers*	*Patients*				
	
	*P1*	*P2*	*P3*	*P4*	*P5*
M1	0.9	0.8	0	1.3	0.3
M2	0.8	0.5	0	1.5	0
M3	0.4	0	0	−0.1	−0.3
Mean	0.7	0.43	0	0.97	0.2
Chi square	0.7	0.8	0.8	1.2	0.9

**Table 2:** Indices calculated from the dose volume histogram of target and normal tissue of different patients

*Indices*	*Patients*					*Mean*	*SD*
			
	*P1*	*P2*	*P3*	*P4*	*P5*		
CI^a^	0.83	0.87	0.79	0.77	0.8	0.81	0.039
EI^b^	0.51	0.66	0.76	0.3	0.52	0.55	0.174
HI^c^	0.61	0.74	0.68	0.55	0.69	0.65	0.074
OI^d^	0.04	0.04	0.02	0.06	0.03	0.04	0.015
COIN^e^	0.51	0.61	0.4	0.55	0.55	0.52	0.078

a CI-Coverage index

b EI–External volume index

c HI–Relative dose homogeneity index

d OI-Overdose volume index

e COIN–Conformal index

**Figure 5A:**
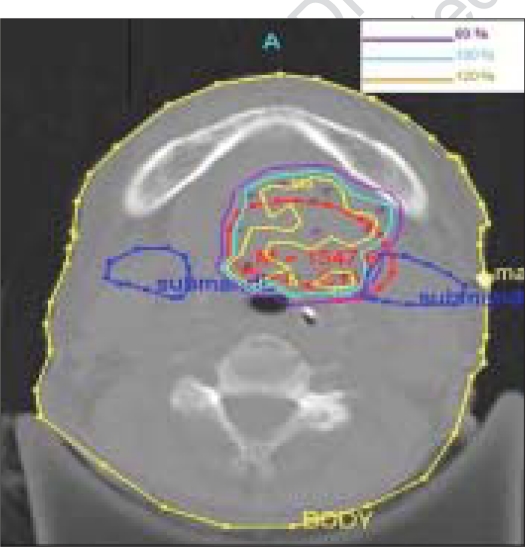
Mapped dose distribution on the axial plane

**Figure 5B:**
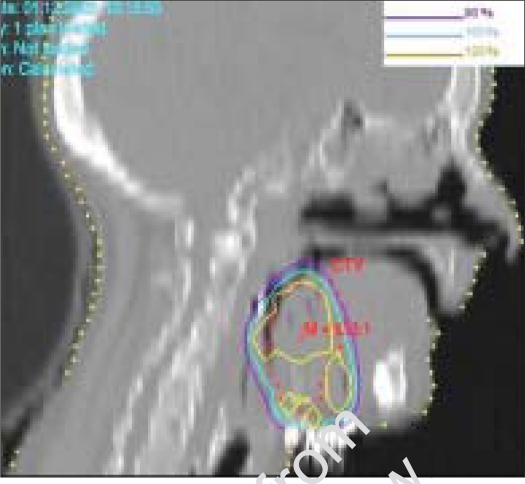
Mapped dose distribution on the sagittal plane

**Figure 5C:**
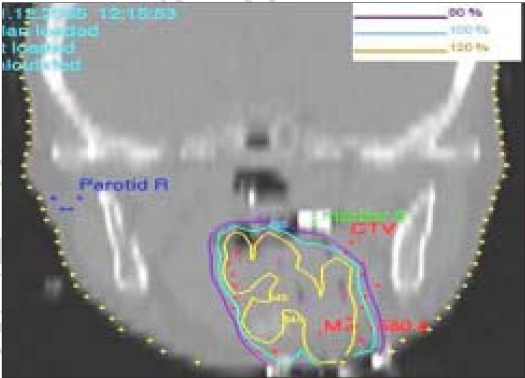
Mapped dose distribution on the coronal plane

**Figure 6:**
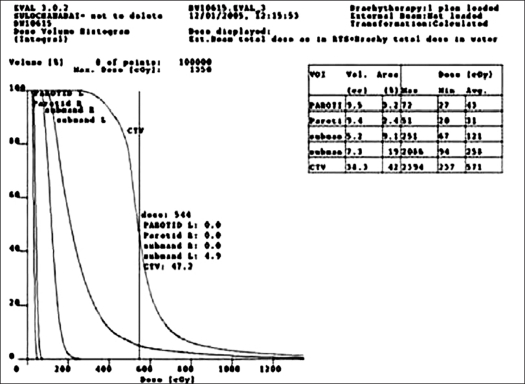
Cumulative dose volume histogram (DVH) of the defined structures on the CT dataset

## Discussion

Unlike other clinical sites, head and neck implant still remains one of the most complicated procedures because of the complex relationship of anatomical structure with disease. Since the inception of interstitial implants at our center in the year 1980, dosimetry had been carried out using a set of orthogonal radiographs. The dosimetric outcome of the implant was evaluated qualitatively by observing the isodose distribution in three orthogonal planes of the implanted geometry. However, this dosimetric system did not provide a three-dimensional relationship between the implanted volume and the anatomic boundaries of the target volume. As a result, implant quality estimated using Anderson DVH might not necessarily correlate with tumor coverage. Hence, to enable a more clinically realistic evaluation of the implant dosimetry, anatomy (CT) based dosimetry need to be adopted. A very limited number of articles on CT-based dosimetry of interstitial implants have been reported; mostly, these have been for breast cancer.[1–4] At the time when 3D CT-based TPS was not available for BT, Vicini *et al*.[1] retrospectively translated the source positions and dwell times planed on 2D BT TPS (Nucletron) using radiographs onto the CT data set of the same patient on a different 3D TPS (ADAC Pinnacle) for quantitative evaluation. In their study, standard needle template implant was used in breast cancer, and the transformation was carried out using in-house developed software. In our proposed technique, more complicated implant geometry was tested using a single TPS and coordinate transformation method. Moreover, in most of the reported studies, only few dosimetric quality parameters related to coverage and homogeneity were addressed. Though modern TPS's support reconstruction of catheter in multiple CT reconstructed plane, our Plato TPS did not have this option at the time of this study.

The maximum variation between the corresponding reference markers during the coordinate transformation was observed to be 1.5 mm. While performing coordinate transformation, out of many reference internal and external markers, those which resulted in least variation were selected for the final mapping. The projection of the catheters represented by a train of active sources was verified on the CT images. A maximum dosimetric inaccuracy of 1.6% was reported by Vicini *et al*.[1] as a result of ±2 mm mismatch in the implant template when transformed from x-ray to CT data set.

The mean CI (0.81) estimated in our study was less than 0.95, as reported by Major *et al*.[8] for ideal implant geometry. However, our mean CI is in agreement with the value suggested by Baltas *et al*.[7] Das *et al*.[2] also reported a CI value of 0.96 in a series of early-stage breast cancer patients treated with image-guided interstitial implant technique for accelerated partial breast irradiation. In their study, graphical optimization was used interactively to achieve higher dose conformality to the target volume. However, the interactive optimization may perturb the homogeneity and lead to an increase in OI. The data from our dosimetry may not be comparable directly with other's data as most of the studies were reported for breast cancers. Higher values of EI in our study indicate larger volumes of normal tissues irradiated by the reference dose. This prescription dose volume to surrounding normal tissue could be avoided if the active source-loading is performed based on the target volume delineated from CT rather than deciding the active length from the radiograph. The value of EI reported by Major *et al*.[8] for idealized implants ranged from 0.17 to 0.44 for various dosimetry systems.

Our mean value of HI (0.65) is in good agreement with the finding of Major *et al*.[8] They reported mean HI of 0.68 for an ideal implant geometry using stepping source dosimetry system. The maximum estimated value of OI is 0.06, and it is well below the reported value (0.11-0.13).[8] The lower value of COIN in this study is due to larger normal tissue irradiated by the prescription dose. Conformal dosimetry system (CDS) developed by Baltas *et al*.[7] aimed to achieve a COIN value above 0.64. However, COIN values reported by several authors ranged from 0.48 to 0.76.[4,7]

## Conclusion

The proposed technique of ‘CT image’-based evaluation of a radiograph-generated plan for complex interstitial implant is a promising approach in the absence of multiplane catheter reconstruction software. This technique is feasible and accurate even for the most complicated implant geometry. It also allows the physicist and physician to evaluate the plan both qualitatively and quantitatively to achieve desired conformity as well as homogeneity without compromising on reconstruction accuracy. Dose volume indices derived from CT data set are useful for evaluating the implant as well as comparing different brachytherapy plans. COIN index is an important tool to assess the target coverage and sparing of normal tissues in brachytherapy.
